# Biomechanics of Heading a Soccer Ball: Implications for Player Safety

**DOI:** 10.1100/tsw.2001.56

**Published:** 2001-08-08

**Authors:** Charles F. Babbs

**Affiliations:** Department of Basic Medical Sciences, Purdue University, West Lafayette, Indiana 47907-1246, USA

**Keywords:** acceleration, brain, concussion, football, head, heading, injury, player, safety, soccer, trauma

## Abstract

To better understand the risk and safety of heading a soccer ball, the author created a set of simple mathematical models based upon Newton's second law of motion to describe the physics of heading. These models describe the player, the ball, the flight of the ball before impact, the motion of the head and ball during impact, and the effects of all of these upon the intensity and the duration of acceleration of the head. The calculated head accelerations were compared to those during presumably safe daily activities of jumping, dancing, and head nodding and also were related to established criteria for serious head injury from the motor vehicle crash literature. The results suggest heading is usually safe but occasionally dangerous, depending on key characteristics of both the player and the ball. Safety is greatly improved when players head the ball with greater effective body mass, which is determined by a player's size, strength, and technique. Smaller youth players, because of their lesser body mass, are more at risk of potentially dangerous headers than are adults, even when using current youth size balls. Lower ball inflation pressure reduces risk of dangerous head accelerations. Lower pressure balls also have greater “touch” and “playability”, measured in terms of contact time and contact area between foot and ball during a kick. Focus on teaching proper technique, the re-design of age-appropriate balls for young players with reduced weight and inflation pressure, and avoidance of head contact with fast, rising balls kicked at close range can substantially reduce risk of subtle brain injury in players who head soccer balls.

### INTRODUCTION

Modern medicine abounds with examples of previously unappreciated harmful effects of repeated subtle injury to the body. Familiar examples include lung cancer caused by cigarette smoking, skin cancer caused by sun exposure, auditory nerve damage or deafness caused by loud noises, and brain damage caused by alcohol ingestion[1]. In the particular case of mechanical injury to the brain, shaken baby syndrome is now recognized as an important ill effect of head acceleration previously thought to be tolerable. Pugilistic dementia in prizefighters subjected to repeated blows to the head is a recognized medical condition[2]. Even mild concussions, when repeated, are now well documented to produce severe and lasting brain damage[3,4].

The sport of soccer (known as football outside the United States) is unique in that the ball can be directed deliberately and purposefully with the head—a play that is termed “heading” the ball. A series of studies reported from Norway has suggested that brain atrophy upon CT scanning[5], electroencephalographic changes[6], and subtle deficits in memory, concentration, planning, and alertness[7,8] occur in long-time soccer players that can be related to the number of balls headed. Soccer players may also suffer repeated mild concussions from collisions with other players, from high kicks, or from falling to the ground, which might possibly explain brain abnormalities in the absence of injurious effects of heading the ball[9,10]. Interestingly, Barnes[10] and Boden[11] studied the incidence of concussions in players from contact with objects other than the ball, such as the ground, a goal post, or another player. Both studies found the incidence of one such non-ball-related concussion to be 1 per 20 years of active playing*. This incidence is much too low to explain repeated or lasting brain injury in typical soccer players[12], especially when two thirds of such concussions were mild, Grade I concussions, characterized by confusion without amnesia[10]. Hence one must seriously consider head-ball contact as a possible cause, either in heading accidents, in which balls strike players unaware, or in normal heading that occurs in the routine course of a playing career.

Matser and coworkers[8,13] found that Dutch amateur and professional soccer players performed significantly worse in tests of memory and planning than did control runners and swimmers. A study of high level youth players in the United States found 40% of those who headed the ball frequently had impaired IQ scores[14]. In contrast, a well-conducted study of U.S. National team players with a long history of heading[15] found no evidence of brain injury compared with track athletes unexposed to heading. The issue remains controversial and of substantial concern to parents of younger players who take up the sport early and may quickly advance to higher competitive levels where heading is an encouraged and expected part of the game[16]. These same players will be exposed to a lifetime of play and could be at risk of cumulative acceleration-related brain injury. Considering the estimated 200 million active soccer players worldwide[13,17], even a small percentage risk of permanent brain injury would have serious public health implications. More research has been encouraged by many authorities[10,13,17,18,19] to resolve outstanding disagreements in the scientific literature concerning the safety of repeated heading. In particular, Jordan et al. have called for further biomechanical studies “to assess not only the forces in headings, but the influence of various mechanisms of heading on impact forces”[15].

The present study is dedicated to the proposition that more detailed knowledge of the mechanics of head-ball impacts can help substantially to resolve whether such impacts are safe or harmful. Better knowledge of the physics involved can lead to recommendations to minimize risk of injury and, with luck, simultaneously increase the enjoyment and artistry of the game, both for players and for spectators. The analysis presented here is intentionally derived in detail from first

* Combining data for men and women, Boden found the probability of concussion to be 0.0005 per exposure (i.e., a game or practice). At 100 games or practices per year, this is a concussion rate of 0.5 concussions per 10 years or one concussion per 20 years. Barns found that the odds are 50% that a man will sustain a concussion within a 10-year period, which gives an expected value of 1 concussion per 20 years.

principles of physics (Newton's second law of motion) using simple models to emphasize the fundamental nature of the results. The methods can be replicated by anyone worldwide with knowledge of calculus and the most rudimentary and inexpensive scientific equipment: a stopwatch, meter stick, and balance. This theoretical approach is intended to be complimentary to, and not competitive with, observational and experimental approaches to the problem.

In the present analytical study the player's body is regarded as a slab-like mass moving forward at a known velocity at the moment of contact. The ball is regarded as a spherical mass approaching in the opposite direction, cushioned by the pressure inside the ball, and damped by viscoelastic indentation of its cover. The problem is simplified by considering horizontal motion only, that is, motion in the "x-direction" over the ground. In standing or jumping headers the players are essentially upright and the force of the ball accelerates the head backward horizontally. In diving headers the body is laid out prone and the force of the ball accelerates head and body horizontally. Accordingly, the horizontal component of acceleration is regarded as most important from the standpoint of safety.

Factors influencing horizontal acceleration include the mass, size, horizontal speed, and inflation pressure of the ball, and the effective mass and speed of the player. Ranges for the masses, diameters, and recommended inflation pressures for youth and adult size soccer balls are available from the manufacturers and from the Laws of the Game[20,21]. Horizontal ball speeds are obtained by observation of actual games with suitable accounting for the effects of air resistance or drag on the motion of the ball. The spring constant for the ball model is a simple function of its inflation pressure. The damping coefficient of a ball is determined from measurements of the height, $h_1$, to which a ball bounces for the first time from a hard surface when dropped initially from height, $h_0$.

With the above information Newton's second law of motion (Force = mass x acceleration) may be used to compute head acceleration during impact for a wide variety of game scenarios. These include large and small players and realistic ball trajectories, including goal kicks, corner kicks, crosses, clearances, and shots. It is also possible to simulate poor or good technique by substituting a small or large "effective mass" of the player. Computed head acceleration versus time curves are then compared with known safe accelerations produced by normal human activities such as jumping or head nodding, which the human body has been designed to withstand. Head acceleration and impact duration data also are compared with the "head injury criterion" (HIC) for known harmful accelerations causing serious injury as defined in the motor vehicle crash literature[22,23,24]. In the end, safe conditions for heading can be defined that produce accelerations far from harmful levels causing head injury and close to the safe levels of normal daily activity.

### RESULTS

#### Horizontal Ball Velocities

Fig. 1 illustrates average trajectories calculated for lofted balls observed in games involving youth players ages 10 and 14, and adult men and women at the skilled amateur and professional levels. These complete trajectories were calculated using the equations of motion for spherical projectiles slowed by air resistance (Appendix 4), given the time of flight and distance traveled by balls observed in actual games. In the figure data points indicate equal time points, separated by 0.1 sec, hence the distance between data points on the trajectory curves is related to a ball's speed.

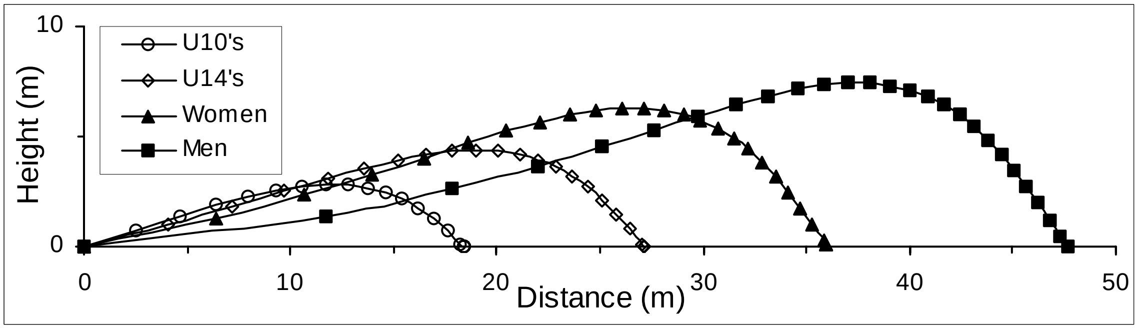

**FIGURE 1.** Calculated average trajectories of lofted balls observed in soccer games. Curves show nonparabolic shapes because of air resistance. Data points are equally spaced in time, 0.1 sec apart. U10's are under 10 ½ year-old players. U14's are under 14 ½ year-old players.

As the size and strength of the players increases the distances covered and initial velocities of balls increase, as expected. However, air resistance, which is related to the square of velocity, also increases and has a longer time to act as balls travel farther. As a result, the horizontal component of velocity, which is relevant to heading safety, becomes similar for all types of lofted balls on the descending limb of flight, regardless of the age and strength of the players. Thus the data points tend to become equally spaced for all types of players on the descending limbs of the trajectories. The spacing of data points for rising balls differs among adult versus youth players. Rising balls kicked by adult players at close range are exceedingly fast, with initial horizontal velocities of 25 to 75 m/sec for adult players, in keeping with previous reports[15,17]. Note that these high velocities are initial values as the ball leaves the foot, not average values.

Fig. 2 illustrates the statistical distributions of horizontal ball velocities during descent at head height for male and female players ages 10 through adult in 16 competitive games. The values range between 1 and 14 m/sec.

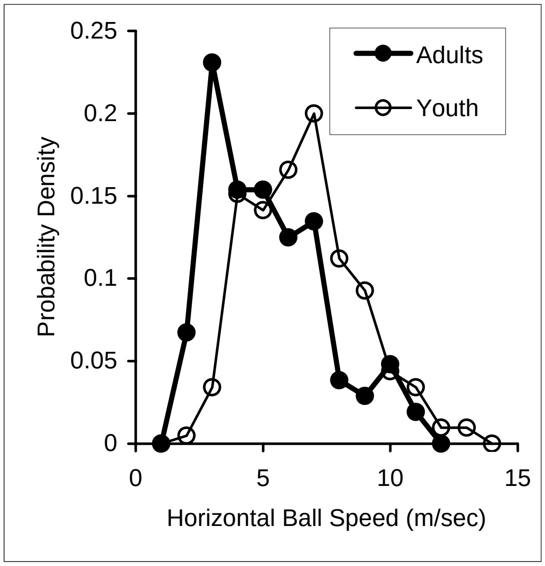

**FIGURE 2.** Distributions of calculated horizontal ball speeds for falling balls at head height obtained from spherical projectile formulas using time and distance data recorded during 16 competitive games. The adult distribution represents 104 balls that could have been headed. The youth distribution represents 205 balls that could have been headed.

Because of the equalizing effect of air resistance, the distributions of horizontal, head-height velocities are similar despite wide variation in the size and strength of the players. A one-way analysis of variance showed no significant difference in descending horizontal, head-height velocities among the age/sex games observed, except for the youth vs. adult categories. Hence only two combined ball speed distributions are shown in Fig. 2. The mean value for adults is 5.7 m/sec, and the mean value for youth players is 7.1 m/sec. Overall, horizontal velocities of “headable” descending balls were slightly greater in youth soccer games than in adult games, even though the initial velocities of balls leaving the foot were markedly less in youth games than in adult games. These counterintuitive differences are a result of the underlying physics involved. They have substantial implications for the safety of heading by smaller youth players.

### Effective Mass of the Player

Upon reflection, one can represent many essential and important features of the biomechanics of heading as a relatively simple one-dimensional problem. Consider a scenario in which a player running due east strikes a ball with the forehead that is moving due west, and consider the horizontal components of head and ball motion. In this case an important result of the present analysis (Appendix 1) is that the player can be regarded as a single east moving “effective mass” that collides with the west moving ball to change its motion. The “effective mass” is the mass that would oppose horizontal acceleration of the head and body to the same degree as would the three dimensional body of the player. The effective mass is defined as $m' = F_x / a_x$, the ratio of horizontal force to horizontal acceleration. The notion of the effective mass of an object with a linearly distributed mass density is well precedented in physics[25,26]. As shown in Appendix 1, the effective mass depends upon the angle, θ, of inclination of the player with respect to the ground. Suppose that the player uses excellent technique, with the neck strong and stiff, so that the head does not wobble backwards significantly. In this case the effective mass of a player inclined at angle, θ, is

$$m' = \frac{m}{2\sin^2\theta} \quad (1a)$$

for a grounded player and

$$m' = \frac{m}{2\sin^2\theta + \cos^2\theta} \quad (1b)$$

for a jumping player. For example, the effective mass for a player standing vertically (θ = 90°, sin(θ) = 1) is one half the body mass for either a grounded or jumping player model. The effective mass for a perfectly horizontal diving header (θ = 0°, feet off ground, sin(θ) = 0, cos(θ) = 1) is 100% the body mass, as expected.

Expressions (1a) and (1b) represent the effective mass of a player using the ideal heading technique, in which the player attacks the ball with neck muscles tensed so that entire body mass is connected to the ball during impact. To model the worst possible heading technique, in which the player is blind-sided or hit unaware by a ball, the mass of the head only is used. The mass of the head alone is in the range of 2 to 5 kg, depending on age and sex[27]. The head mass is much less than one half body weight and therefore much more susceptible to acceleration during head-ball contact than the effective mass of a player using good technique. Values of effective mass intermediate between that of the head and one half the total body mass may be used to simulate mediocre technique, in which the head is allowed to wobble slightly. Using this simple convention to represent a range of complex techniques and the many musculoskeletal linkages between head, neck, spine, and trunk, one may regard the act of heading a soccer ball as a collision between the ball itself and a forehead with a certain effective mass moving in the opposite direction. Youth players have a much smaller effective mass than do adult players, and youth players who have not yet learned to use good technique have a smaller effective mass still. For any particular trajectory of an incoming ball, the effective mass of the player is a major determinate of head acceleration at impact. The greater the effective mass of the player, the smaller is the acceleration of the head.

### Head Acceleration

Fig. 3 illustrates horizontal motion of head and ball as a function of time in a typical adult heading impact. The figure is a plot of the horizontal positions of head and ball as a function of time after their initial contact. These positions were calculated using Newton's second law of motion for a standard size 5 soccer ball colliding with a 70-kg adult player having an effective mass of 35 kg. Time zero on the horizontal axis in Fig. 3 represents the instant of head–ball contact. Position zero on the vertical axis of Fig. 3 represents the point of initial head–ball contact. Positive values of position on the graph indicate forward progress in the direction the player is facing. The position of the ball is reckoned from a point on its usual, nonindented circumference closest to the head. At the instants of contact and of separation the center of the ball is exactly one radius away, hence ball and head positions indicated in Fig. 3 are the same, and the curves cross. When the position of the ball is plotted below that of the head, the head is indenting the ball.

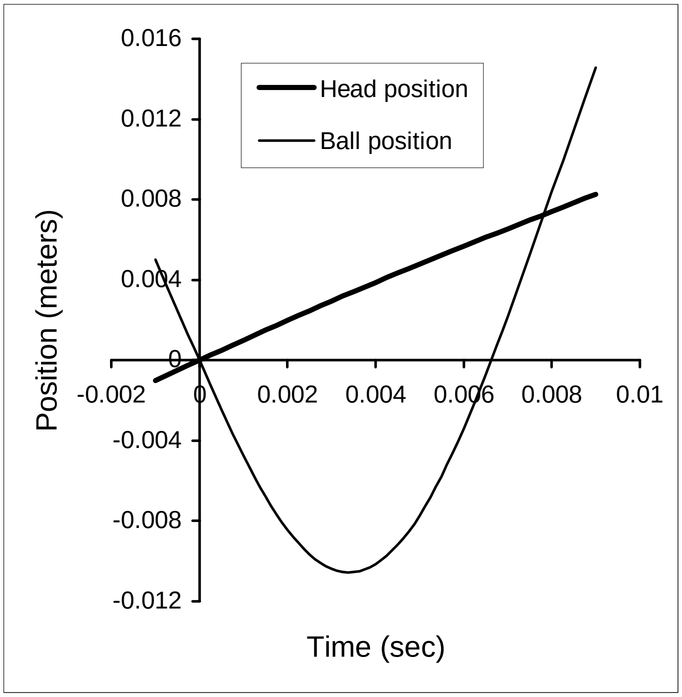

**FIGURE 3.** Time course of positions of head and ball during impact, plotted with respect to the initial contact point. Impact begins at coordinates 0, 0. The effective mass of the adult player in this example is 35 kg, indicating excellent technique.

In the example shown in Fig. 3 the player is moving at 1 m/sec toward the ball, which in turn is moving horizontally toward the player at 5 m/sec. The relative curvatures of the head and ball tracings, indicating acceleration, are related by the ratio of ball to player mass ($a_1/a_2 = -m_2/m_1$), using the effective mass of the player. The shape of the instantaneous head acceleration vs. time curve is shown in Fig. 4. In this coordinate system head acceleration is negative (i.e., backward).

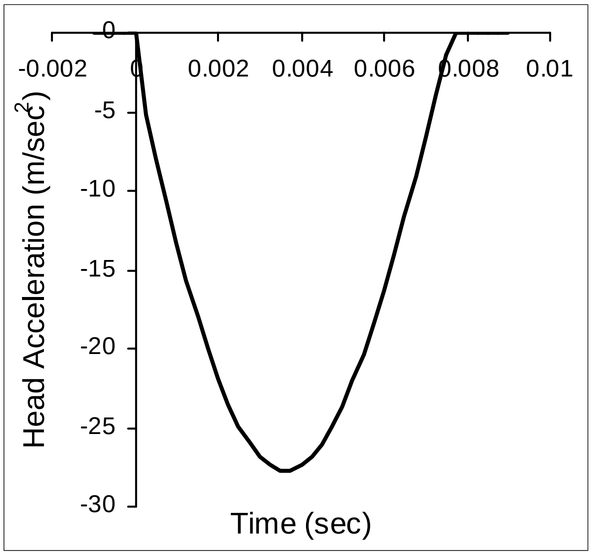

**FIGURE 4.** Instantaneous head acceleration during head–ball impact. Here 10 m/sec$^2$ is approximately equal to 1 *g*. The small deviations from a perfect half-sinusoidal curve are caused by energy dissipation in the cover of the ball. Maximal head acceleration approaches 3 *g*.

The harmful effects of head acceleration are functions of the duration of the acceleration and the mean value of acceleration during this interval[22,23,24]. These values for any particular case can be represented as a point in mean acceleration–duration space (see plots in Fig. 5). To provide a reference for interpretation of heading safety, curves describing the head injury criterion (HIC) for clearly harmful and potentially fatal single impacts (HIC = 1000 $g^{2.5}$-sec) are plotted as the upper lines in Fig. 5 (note log scale). Parallel lines representing a putative safe value of the HIC are plotted toward the bottom. This presumed safe level corresponds to accelerations experienced during normal movement in sports and in daily life (Table 2, Methods) and corresponds to an HIC value of 0.2 $g^{2.5}$-sec.

The data points in Fig. 5 represent results of Monte Carlo simulations, in which head-ball impacts were computed for 100 randomly selected ball trajectories and effective player masses. For each impact the mean head acceleration during impact and the total duration of impact are indicated by the position of the data point in acceleration–duration space. One hundred point Monte Carlo simulations are shown for each of three ball inflation pressures ranging from 0.3 to 1.1 atmospheres (soft, medium, and hard balls). In this way the wide variability of head acceleration experienced during heading under normal playing conditions can be illustrated. The top plot represents a model of an adult player weighing 70 kg who is heading standard adult, size 5 balls. The bottom plot represents a model of a smaller youth player weighing 40 kg who is heading smaller size 4 balls.

An average adult header performed with good technique produces an impact lasting 0.0085 sec with mean head acceleration of about 2.4 g and an HIC of 0.07 $g^{2.5}$-sec. This average impact for properly executed adult heading is below the putative safe threshold of 0.2 $g^{2.5}$-sec. However,

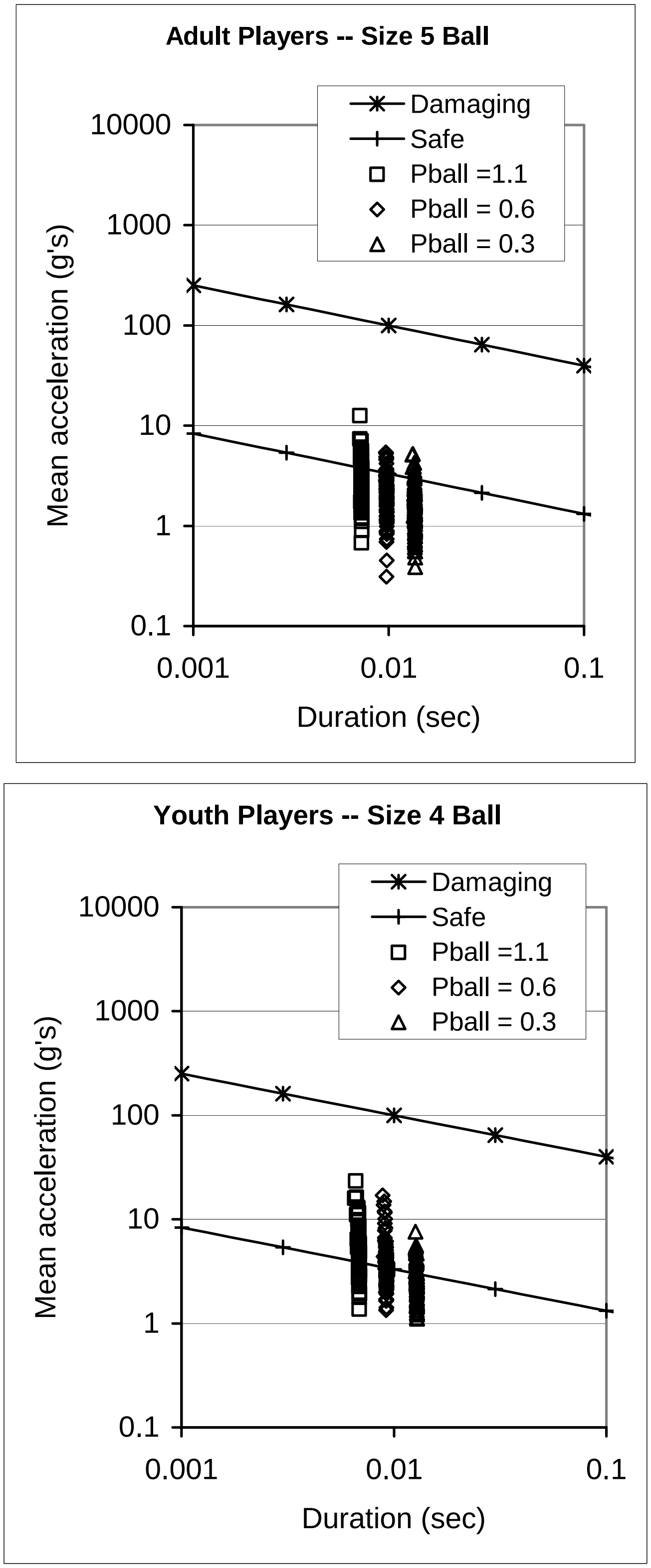

**FIGURE 5.** Characterization of heading impacts by comparison with clearly safe and clearly damaging fractions of the head injury criterion. For each ball inflation pressure (0.3, 0.6, or 1.1 atm), 100 randomly selected combinations of the player's effective mass and the ball's horizontal velocity were used to compute the plotted data points.

heading impacts are not invariably safe, because the HIC varies as a 2.5th power function of head acceleration. Consequently, small increases in head acceleration produce large increases in the HIC.

In the case of adults using a normal adult size 5 ball inflated to any of the three pressures, most combinations of ball speed and technique produced impacts with HIC values less than the putative safe level of 0.2 $g^{2.5}$-sec. One outlier for a high, but legal pressure ball was clearly above the limit, suggesting that in a life-long soccer career a few such questionably safe headers may occur during normal play. A small proportion of headers had HIC values between 0.2 and 0.5, only slightly above the threshold of normal activity. Thus normal heading by adults would appear to be nearly as safe as head nodding or jumping according to the head injury criterion.

The situation for youth players, however, is rather different. Here the effective body mass of youth players was selected at random from a normal (Gaussian) distribution with mean 15.4 kg and standard deviation 4.6 kg. These values are 40/70 times those used for adult players, corresponding to a mean youth player weight of 40 kg compared to a 70-kg adult. This body weight is that of an average 10½-year-old girl or an 11-year-old boy. The horizontal ball velocity was selected at random from a Gaussian distribution with mean of 7.1 m/sec and standard deviation 2.2 m/sec, which represents closely the observed distribution of horizontal ball velocities for youth players, ages 9 to 13, in Fig. 2. In addition, weight and circumference data for a smaller, size 4 soccer ball were used in computing head accelerations for youth players. The overall range of possible accelerations during heading of legally inflated balls is clearly greater for youth players than for adults. Use of a smaller, size 4 ball does not adequately compensate for the smaller effective mass of younger players. For 0.6 to 1.1 atm inflation pressure, the acceleration distributions for size 4 balls fall on both sides of the presumed safe level of 0.2 $g^{2.5}$-sec. The higher accelerations for youth players are in the range 15 to 20 $g$ when unlucky combinations of high ball velocity, high inflation pressure, and bad technique occur.

### Reduced Pressure Balls

One easy and obvious modification of the ball to increase heading safety, especially for young players, is shown in Fig. 6. This chart illustrates combinations of total body mass and ball pressure meeting the “safe” level in Fig. 5 with HIC = 0.2.

Smaller, lighter weight individuals can head balls safely when ball inflation pressure is reduced. Reduced pressure balls, inflated to 0.3 atmosphere or 5 PSI, are softer than standard balls. They have “reduced bounce” on a hard surface, but not obviously “flat”. They are similar to older practice balls used by many teams that are not meticulously checked for pressure and tend to loose air over time. Moreover, as indicated by the bottom curve in Fig. 6, youth players 30 kg in weight (7 to 12 years old) can head a soft rubber playground ball safely in practice.

### Touch and Playability

A further advantage of reduced inflation pressure for standard soccer balls can be demonstrated directly by the mathematics of head and ball acceleration. Consider the ball impactor in expressions (4) and (5) (see Methods) to be the instep of the foot with an area of 100 cm$^2$, rather than the head. Next, regard $v_1$ as foot speed (e.g., 2 m/sec) and $v_2$ as ball speed (e.g., zero). The result is a simple model of foot–ball impact during practical play. In this model two measures of touch and playability of the ball are the contact time and the maximal contact area at peak indentation. Greater contact time and greater contact area mean greater control over the ball or greater “touch”. These variables are illustrated as a function of inflation pressure, together with ball speed leaving the foot in Fig. 7.

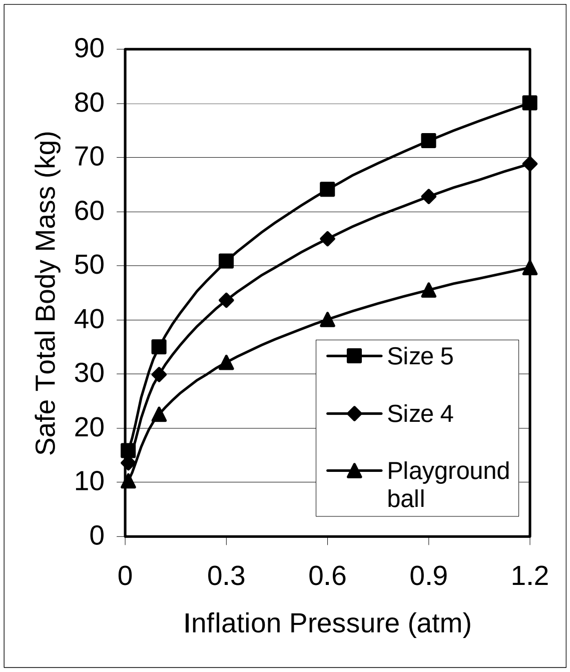

**FIGURE 6.** Combinations of total body mass (2x the effective mass) and ball pressure meeting the "safe" criterion of Fig. 5 for heading with proper technique. Adult (size 5) and youth (size 4) soccer balls are compared with a 226-g rubber playground ball. Smaller, lighter weight children can head lower pressure balls safely. Greater body mass is required for clearly safe heading of over-inflated balls. Horizontal ball speed toward the head is assumed to be 12 m/sec.

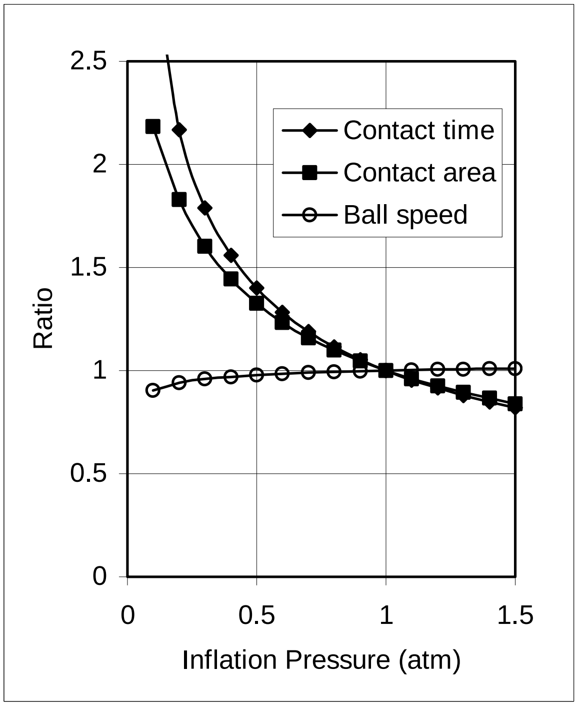

**FIGURE 7.** Relative changes in touch factors and ball speed as a function of ball inflation pressure. As inflation pressure is reduced, a slight reduction in ball speed leaving the foot is accompanied by much larger increases in contact time and contact area. Values relative to those at 1.0 atm inflation pressure are plotted.

Measures of touch increase as inflation pressure decreases. The penalty in reduced ball speed with reduced inflation pressure is small, 4% at 0.3 atmospheres. Thus the shooter's touch and control are theoretically much improved with softer balls, while the goalkeeper's time to react (time of flight) is increased only slightly. In this sense, softer balls cannot only improve heading safety, but quite possibly may also improve goal scoring and excitement of the game by providing strikers with more control to place shots beyond the goalkeeper's reach as well as by enabling greater passing accuracy. It is important to consider the physical factors related to touch and control, because many soccer players believe that softer balls are undesirable because they are slower than hard, over-inflated balls. The importance of touch factors, however, is rarely considered.

### Monte Carlo Simulation of an Entire Playing Career

In a lifetime career of normal soccer playing, a few unlucky combinations of high-speed balls and poor technique may well produce occasional impacts with disturbing values of the HIC. To explore this possibility one may easily conduct a Monte Carlo simulation of a lifelong playing career. Ten thousand headers would be taken in 20 years, assuming averages of 20 weeks of play per year, 5 practices or games per week, and 5 headers per game or practice. In performing such a simulation, the author selected ball velocities and techniques at random from realistic sampling distributions, chosen to reflect both youth and adult play. Means and standard deviations of these distributions were an effective mass 24 ± 10 kg (but never less than 3 kg for head mass) and horizontal ball velocity 6.0 ± 2.5 m/sec. Ball inflation pressure was 1.0 atm. The simulation predicted 64 unlucky headers with HIC's in the range of 30 to 100 (3 to 10% of the single impact danger level). Such impacts may well represent harmful subconcussive blows or low-grade concussions, despite the general use of good technique. When ball inflation pressure was reduced to 0.3 atm, the simulation predicted only 1 header with an HIC between 10 and 30.

### Accidental Heading

In addition to normal play, just described, a further cause of dangerous head-ball impacts during a playing career is "accidental heading", when balls kicked with full force at close range strike players. In such accidents players may be caught unaware and therefore not react quickly enough to protect themselves[11]. Such accidental heading can occur when players in a defensive "wall" are struck by a free kick at 10 yards range, or when players are accidentally hit while fetching balls near the goal during shooting practice. Such accidents may occur several times in a player's career and provide a partial explanation for subtle brain injury described in adult amateur and professional players. Models of accidental heading are easily derived from data like those presented in Fig. 1. A rising ball kicked with average force 10 meters away may have a horizontal speed of up to 50 m/sec. If such a ball were to strike an unprepared player with an effective head mass of 5 kg, the mean head acceleration would be 106 $g$ with an HIC of 844 $g^{2.5}$-sec. Such situations are clearly concussive and dangerous. Asami and Nolte measured speeds of regulation soccer balls kicked by professional players ranging from 28 to 34 m/sec[28]. If speeds of 27 to 54 m/sec are used, as reported by Jordan[15], for balls inflated to 1.0 atmosphere, values of HIC range from 190 to 1020 $g^{2.5}$-sec. For balls inflated to 0.3 atmosphere, the corresponding HIC values range from 70 to 380 $g^{2.5}$-sec.

### DISCUSSION

Newton's second law of motion implies a great deal about the safety of heading in soccer games. Certain mathematical inevitabilities about the interaction of masses moving at known velocities govern the acceleration of a player's head connecting with a high ball during a game or practice. Balls move much faster than players in a soccer game, and so their speed and mass largely determine the forces acting upon the head. The horizontal component of ball velocity is the component of relevance in heading safety. Our observational data show that the range of horizontal speeds of balls in a position to be headed is relatively constant in actual games, despite large variations in the size and strength of the players. In turn, the head is accelerated in inverse proportion to the effective mass of the player, which depends upon the player's body size and technique. One major variable under control of coaches and sports officials is the pressure inside the ball. Lower-pressure balls cause less head acceleration over longer contact times than do higher-pressure balls. This flatter acceleration vs. time profile produces lower values of the HIC than shorter but higher intensity accelerations. The longer contact time and larger contact area of low-pressure balls played with either head or foot also give players more control over where the ball will go. Lower-pressure balls may thus improve both the safety and enjoyment of the game. To realize such benefits, however, it will be necessary to change the negative attitudes of many soccer players regarding softer balls by emphasizing their greater safety and "touch". We should argue against hard ball macho.

The goal of the present analysis was to capture the essence of the physics of soccer heading. Simplification of the problem by neglecting acceleration in the vertical dimension and the use of the effective mass concept to account for varying body size and rigidity of the neck leads to mathematics describing two masses connected by a linear spring and damper in one dimension. Use of such a simple model as an initial approach to the biomechanical problem is similar to that employed by Crisco, Hendee, and Greenwald[29] in studying impacts between baseballs with the human head and chest. The use of the head injury criterion as a reference side-steps the complex issue of exactly which mechanical events lead to closed head injury including lysis of axons and capillaries in the brain[30,31] by substituting the descriptive formula of the HIC for detailed mechanical interactions. A more complex model in three dimensions incorporating a more realistic rendering of the human body might very well produce additional insights. Nevertheless, real concerns about the safety of normal and accidental head-ball contact over a playing career are legitimately raised by this initial analysis.

The virtues of the present mathematical modeling approach are highlighted by the recent report of Naunheim and coworkers[32]. These investigators measured acceleration within the helmets of high school hockey and football players during impacts in actual game play using an accelerometer placed at the vertex of the helmet immediately adjacent to the player's head. They also measured peak acceleration during head impacts in high school-level soccer players who headed soccer balls while wearing an instrumented football helmet. The values they recorded, averaging 55 $g$ peak acceleration (539 m/sec$^2$), are over an order of magnitude greater than those reported in the present study. This author believes the discrepancy is due to the relatively low mass of the helmet itself, which is coupled by foam rubber to the mass of the head. Those who have worn and played in an American football helmet know that the helmet can move a short distance with respect to the head during an impact. This feature gives the helmet the ability to absorb energy and protect the head. The ratio of helmet acceleration in the study of Naunheim and coworkers to player acceleration in the present study (539/27 = 20) corresponds roughly to the ratio of player effective mass to helmet mass (30 kg/ 1 kg = 30), as would be expected for these brief impacts. The comparison of our two studies highlights the difficulty of measuring cranial acceleration during brief (10 msec) head-ball impacts in actual soccer games, and perhaps illustrates the utility of the present mathematical approach.

Fortunately, the intensity and duration of a single impact required to produce concussion or neuronal injury in the brain have been reasonably well defined in terms of the head injury criterion or HIC. This index is used in evaluation of safety helmets and automobile crash restraints. The present research demonstrates that with proper technique typical normal heading by adult players generates HIC values less than 0.1% of that required to produce brain injury in a single impact. Younger players, however, are more at risk of dangerous ball contact than are adult players, even when a smaller size 4 ball is used. Young players have less effective body mass and less strength and practice using good technique. Moreover, the balls they head are slowed less by air resistance and have slightly greater mean horizontal velocity than balls headed by adults. Routine headers taken by youth players generate HIC values up to 1% of that required to produce brain injury in a single impact (Fig. 5). A concern arising from the seminal work of Cronwall and Wrightson[3] is that repeated concussions produce substantially more severe and prolonged symptoms than do isolated single blows to the head. It is reasonable to suppose that repeated subconcussive blows could have a significant cumulative effect. Accordingly, a player who repeatedly heads the ball — especially accidentally — may risk cumulative brain damage[9,14].

When the neck is loose and the head is allowed to wobble the effective mass of the player approaches the mass of the head. This is the most dangerous situation, producing the highest head accelerations, as can occur when the player is hit unexpectedly (blind-sided) or gives a half-hearted effort.* In practice players should not hang around the goal area when others are taking practice shots to reduce chances of being blind-sided by a ball. Only one striker at a time should shoot against a live goalkeeper, else the keeper could be struck by one ball while watching another (a common problem). Anyone heading a rising ball that has not had time to be slowed by air resistance may suffer potentially damaging brain accelerations. Moreover, a reaction time for such close range impacts is short. Hence the player may not be able to muster sufficient effective mass. One solution to minimize such dangerous headers would be a rule change making heading a rising ball "dangerous play", punishable by a free kick. Thus players would be strongly encouraged to "duck" in these dangerous situations which, from a medical viewpoint, is the better part of valor.

Paradoxically, on the other hand, high balls that seem dangerous really are not. Long times of flight allow air resistance to slow horizontal velocities. As shown in Fig. 1, the higher the ball in practical games the less the horizontal velocity, which is the component of consequence in heading safety. Moreover, diving headers, which may look and seem dangerous, are from the standpoint of the brain relatively safe, because there is more effective body mass in the horizontal dimension, i.e., the effective mass, $m' = m/(2\sin^2\theta + \cos^2\theta)$ approaches $m$, as $\theta$ approaches zero.

### Steps Toward Safety

Minor and prudent adjustments that could be made in the way the game is practiced and played to increase head safety, especially for smaller and younger players, include the following:

1. More extensive use of softer, under-inflated balls, especially during intensive practice;
2. Re-design of youth size balls (sizes 3 and 4) to be lighter in weight with lower inflation pressures, more fully compensating for the smaller in effective mass of younger players;
3. Greater emphasis on proper technique with maximal body mass "behind" the ball, strong and stiff neck;
4. Routine use of neck strengthening exercises during training of players;
5. Training of players to avoid dangerous situations in which they may be struck by rising balls or blind-sided (hanging around a goal during shooting practice), including rule changes to make heading a rising ball at close range "dangerous play".

## METHODS AND PROCEDURES

### Approach

In this study a suite of simple mathematical models was created to describe head acceleration as a function of time during heading of the ball. The problem is simplified by considering horizontal

* A soccer ball has unlimited "reach" unlike a boxer, so moving the head backward with the ball is not a good strategy, as it can be in boxing.

motion only, that is, motion in the "x-direction" over the ground. The horizontal component of acceleration is regarded as the dominant vector of head acceleration. Nomenclature for the models is given in Table 1. This theoretical, biomechanical approach is intended to compliment sophisticated experimental studies. It can yield insights difficult to obtain with accelerometers, high-speed videotape, and human subjects. In theoretical studies one can explore the limits of safety without risk of harming human subjects. One can also do Monte Carlo simulations to explore rare events of unusually high risk. In the analysis some reasonable approximations are utilized to keep mathematics tractable. Errors introduced by the approximations, however, are small, on the order of 1 to 5%. Such inaccuracies are negligible with respect to wide variation in playing conditions, ball speeds, angles, player size, weight, and technique that are present in actual soccer games. The goal is to capture the essence of the phenomena that lead to worrisome head accelerations and to study the key variables involved.

**Table 1**
**Nomenclature**

| Symbol | Definition | Units or value |
|---|---|---|
| A, B, C, D, E, a, b, c, z, $\lambda$ | Defined constants | various |
| $\beta$ | Air resistance coefficient | Nt/m$^2$/sec$^2$ |
| e | Base of natural logarithms | 2.718 |
| $\varepsilon$ | Small value | various |
| F | Force | Newtons |
| f | Frequency | sec$^{-1}$ |
| g | Gravitational acceleration of falling body at Earth's surface | 9.80 m/sec$^2$ |
| h, y | Vertical component of ball trajectory | meters |
| k | Spring constant | Nt/m |
| ln | Natural logarithm | dimensionless |
| m | Mass of object | kg |
| m' | Effective mass of player | kg |
| $\mu$ | Damping coefficient for energy absorption by ball | Nt/m/sec |
| P | Pressure | atmospheres |
| $\pi$ | Circle ratio | 3.14159 |
| R, r | Radius | meters |
| $\rho$ | Axial density of human body per unit length | kg/m |
| t | Time | sec |
| t* | Time to peak height of ball | sec |
| t**, $t_h$ | Hang time for ball to return to earth | sec |
| $\theta$ | Any angle | radians |
| $\tau$ | Time difference | sec |
| u | Velocity difference | m/sec |
| v | Velocity | m/sec |
| $\omega$ | Angular frequency | radians/sec |
| x, d | Distance | meters |
| $\dot{x}$ | Velocity | m/sec |
| $\ddot{x}$ | Acceleration | m/sec$^2$ |
| Subscripts: | | |
| 0, o | Initial value at t=0 | |
| 1 | Denser, heavier mass (player or Earth) | |
| 2 | Less dense mass (ball) | |

### The Player

It is wrong to consider the head as a mass unconnected to the body, since players with even moderately effective training learn to tense the neck muscles to put more effective mass behind the shot. However, it is also wrong to assume the player's entire body mass is lumped behind the ball during heading. The ball is struck by the standing or jumping player at one end of a long lever arm, which is free to rotate backwards either at the point of contact with the ground or in free space (Fig. 8). In the case of grounded players it is useful to the regard the player as a slab-like mass that is free to rotate at the point of foot contact with the turf. In the case of jumping players it is useful to regard the player as a slab-like mass that is free to rotate about its center of gravity.

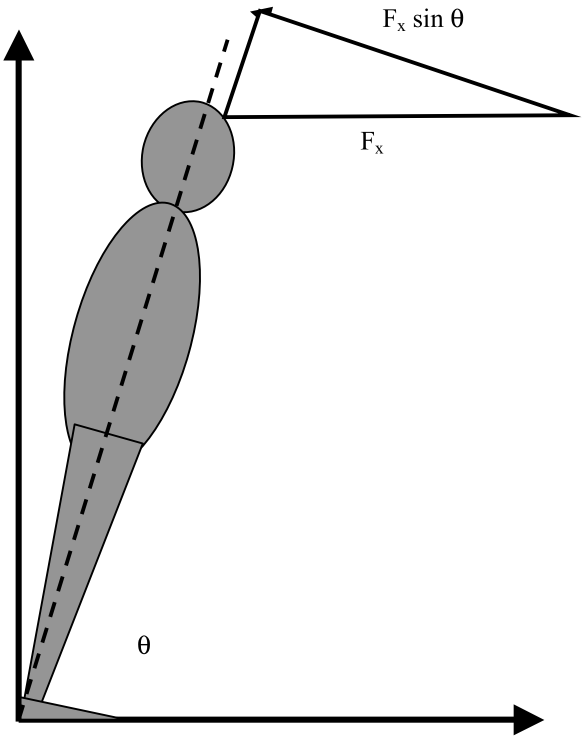

**FIGURE 8.** Model of a grounded player.

The effective mass of the player that opposes horizontal acceleration of the head and body in the x-direction can be defined as $m' = F_x / a_x$, the ratio of horizontal force to horizontal acceleration. The notion of the effective mass of an object with a linearly distributed mass density is well precedented in physics[25,26]. As shown in Appendix 1, the "effective mass" depends upon the angle, θ, of inclination of the player with respect to the ground and the axial distribution of mass along the length of the body. As presented in the Results, the effective mass of a player inclined at angle, θ, is

$$m' = \frac{m}{2\sin^2 \theta} \qquad (1a)$$

for a grounded player and

$$m' = \frac{m}{2\sin^2\theta + \cos^2\theta} \tag{1b}$$

for a jumping player. The effective mass for a uniform density slab at right angles to the ground (θ = 90°) is exactly one half the body mass for either a grounded or jumping player model. The effective mass for a perfectly horizontal diving header (θ = 0°, feet off ground) is 100% of the body mass, as expected.

Most headers are taken at angles from 80° to 100°. Because of the shape of sinusoidal functions, the cosines of such angles are ≈ 0, and the sines of such angles are ≈ 1. In turn, a rule of thumb for effective mass in routine heading performed with ideal technique (neck strong and stiff, no head wobble) is to use one half the body weight. This approximation is true for the mass distribution of a slab. It is also approximately true for a more anatomically realistic model, in which the axial distribution of body weight is not uniform, but varies as a function of height (greater for the abdomen and less for the neck, the partially air-filled chest, and the lower legs). When the mass in such a model is integrated over the entire body length (data not shown), the value for effective mass differs less than 5% from that of a uniform slab.

To model ideal heading technique, in which the player attacks the ball with neck muscles tensed so that entire body mass is connected to the ball during impact, the expression $m' = m/2$ is used. To model the worst possible heading technique in which the player is blind-sided or hit unaware by a ball, the mass of the head only is used. (The mass of the head alone is in the range of 2 to 5 kg, depending on age and sex[27].) Values of effective mass intermediate between that of the head and half the total body mass may be used to simulate mediocre technique, in which the head is allowed to wobble slightly.

### The Ball

One of the pleasing aspects of this research is the simplicity with which the ball can be modeled. Fig. 9 shows a sketch of a spherical soccer ball, partially flattened on one side by impact with the forehead.

The force between ball and head is the product of the internal pressure and the contact area. As shown in Appendix 2, the contact area is approximately equal to $2\pi Ru$ for ball indentations, $u$, that are a small fraction of ball diameter (true for realistic situations). The increase in ball pressure, $P$, caused by reduction in the volume of an indented ball is negligible, hence the force as a function of indentation, $u$, is $F(u) = P \cdot 2\pi Ru$. Thus the ball can be regarded realistically as a mass cushioned by a Hook's law spring with spring constant

$$k = 2\pi RP = C_{ball}P , \tag{2}$$

where $C_{ball}$ is the ball circumference and $P$ is the initial internal pressure measured with respect to ambient atmospheric pressure. Energy absorption or damping by the ball occurs because of flexing or crimping at the edge of the indented area as the head, foot, or ground compresses the ball. Suppose that the amount of energy absorption is proportional to the rate of bending and the circumference of the contact area where bending occurs. Then, as shown in Appendix 2, the small component of force exerted by the ball on the head due to deformation of the cover is simply

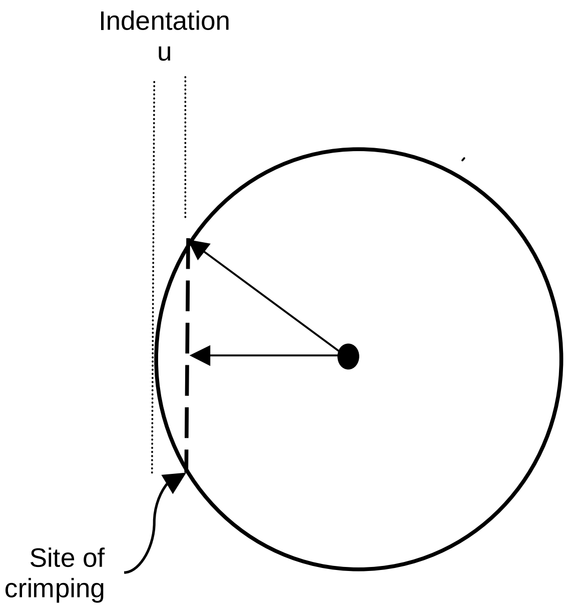

**FIGURE 9.** Analytical model of a soccer ball.

$\mu \frac{du}{dt}$ for a constant damping factor, $\mu$. In turn, the magnitude of the force of the ball upon the head, which is equal and opposite to the force of the head on the ball during impact, is

$$F(t) = ku + \mu \frac{du}{dt}. \tag{3}$$

In expression (3) the $\mu \frac{du}{dt}$ term for soccer balls is small compared to the *ku* term. Hence errors in its estimation, such as the use of a single average value of $\mu$ for all balls, have negligible influence on the overall results. These essential mechanical features of player and ball permit calculation of their mutual accelerations during heading. The damping factor, $\mu$, specifically accounts for energy absorption by the ball during impact.

### The Impact

A most convenient coordinate system for deriving and plotting motions of the head and ball during impact is sketched in Fig. 10. Here the instant of initial contact is defined as time $t = 0$, and the position of initial contact is defined as $x = 0$. The positive direction in the x-dimension is that which the ball will eventually take, i.e., forward. Prior to contact the velocity of the ball is negative and its position is positive.

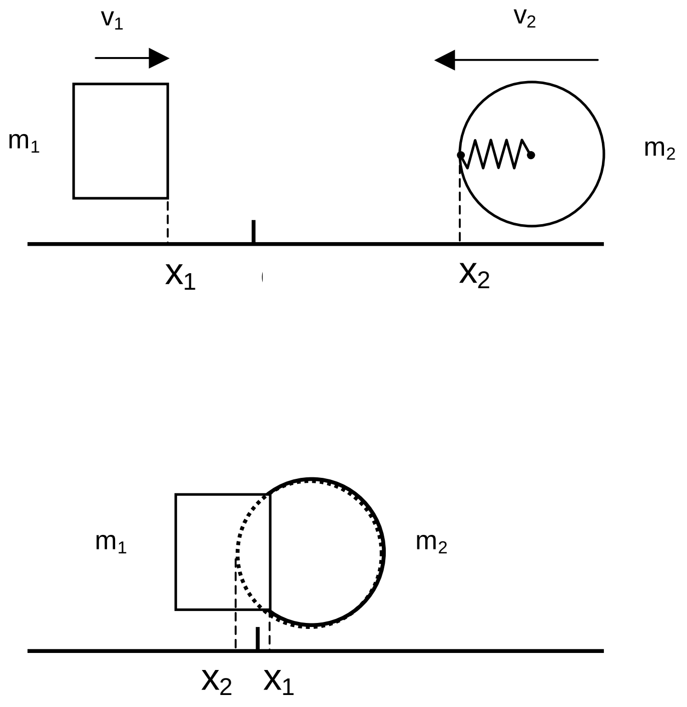

**FIGURE 10.** Coordinate system for describing head–ball impact. Top: before impact. Bottom: during impact. Here the indentation, $x_1 - x_2$ , is exaggerated.

Prior to contact (Fig. 10, top) the velocity of the head is normally positive, moving forward, and the position of the head is negative. During and after impact the velocity of the head normally continues to be positive and the velocity of the ball reverses from negative to positive.

To permit easy charting of ball and head positions at impact on the same set of axes, it is convenient to reckon the position of the ball from a point on its usual, nonindented circumference closest to the head, even when the head is indenting the ball. During indentation the center of the ball is closer to the forehead than one ball radius. At the instants of contact and of separation the center of the ball is exactly one radius away. Hence during impact, when the position of the ball is plotted below that of the head, the head is indenting the ball (Fig. 10, bottom, and Fig. 3).

From Newton's second law of motion as applied to the situation of Fig. 10, we have $F_1 = m_1a_1$, and $F_2 = m_2a_2$, where $m_1$ is the effective mass of the player, $m_2$ is the mass of the ball, and $F_1 = -F_2$. As shown in Appendix 3, combining these fundamental relationships with the force model of expression (3) leads to the equation of motion for the head during impact,

$$x_1(t) = a_1 \sin(\omega t) e^{-bt} + ct, \tag{4}$$

where

$$a_1 = \frac{(v_1(0) - v_2(0))m_2}{\omega(m_1 + m_2)}, \tag{4a}$$

$$b = \mu \frac{m_1 + m_2}{2m_1 m_2}, \tag{4b}$$

$$c = \frac{m_1 v_1(0) + m_2 v_2(0)}{m_1 + m_2}, \tag{4c}$$

and

$$\omega = \sqrt{k \cdot \frac{m_1 + m_2}{m_1 m_2} - \left( \frac{1}{2} \frac{\mu(m_1 + m_2)}{m_1 m_2} \right)^2} \cong \sqrt{k \cdot \frac{m_1 + m_2}{m_1 m_2}} \tag{4d}$$

for realistic values of $\mu$, which are numerically much smaller than $k$. Here k is the spring constant of the ball, $\mu$ is the damping coefficient for the ball, and other variables are defined as in Table 1. Similarly, the equation of motion for the ball during impact is

$$x_2(t) = a_2 \sin(\omega t) e^{-bt} + \ ct, \tag{5}$$

where $a_2 = -\frac{m_1}{m_2} a_1$ and constants $b$, $c$, and ω are the same as for $x_1(t)$.

From the analytical expressions (4) and (5) for positions of the head and ball as functions of time, it is a simple matter to derive the velocity and acceleration of the head and ball by differentiation. Head acceleration, which is of particular interest in the problem of heading safety, is

$$\ddot{x}_1(t) = -a_1[(\omega^2 - b^2)\sin(\omega t) + 2\omega b \cos(\omega t)]e^{-bt}. \tag{6}$$

Before and after impact the accelerations of the both ball and head are zero and of no further interest from the standpoint of head injury.* From expressions (4a) and (6) it can be appreciated that the effect of the speed of the player simply adds to that of the ball. (Note that these are opposite in sign when the ball is coming toward the player, so that the difference $V_1(0) - V_2(0)$ is actually the sum of the absolute values of the player and ball velocities.) If the ball is traveling at 10 m/sec and the player is traveling at 1 m/sec in the opposite direction, the overall effect is the same as if the player is standing still and the ball is traveling at 11 m/sec. That is, the relative speed of the ball with respect to the player is what matters.

### Motion of Balls in the Air Prior to Impact

To determine the possible trajectories and speeds of balls approaching players' heads in soccer games it is helpful to understand the motion of masses in the Earth's gravitational field (1 *g*) in the presence of air resistance or drag. In the absence of air resistance, i.e., in a vacuum, the horizontal component of ball velocity would be a constant, $v_h(0)$, equal to the horizontal velocity

* Air resistance acting on the ball is relevant on time scales on the order of one second and is taken into account in calculation of ball speeds prior to impact. However its effects are negligible on the time scales of milliseconds immediately before and after impact in plots like Fig. 3.

of the ball leaving the foot of the previous player. The vertical component of ball velocity would be $+v_v(0) - gt$, where $g = 9.8$ m/sec$^2$ is the acceleration of a freely falling body near the Earth's surface. From these expressions it would be simple in a vacuum to calculate the complete trajectory of the ball, launched from the ground with initial horizontal and vertical velocity components, $v_h(0)$ and $v_v(0)$. The trajectory is given in terms of the horizontal and vertical components of ball position, namely the distance traveled, $d$, and height, $h$:

$$d(t) = v_h(0)t \tag{7}$$

and

$$h(t) = v_v(0)t - \frac{1}{2}gt^2, \tag{8}$$

where $t$ is the time of flight in seconds.

For a soccer ball, however, air resistance is an important force slowing the horizontal speed substantially toward the end of flight. This effect is significant for understanding the safety of heading. In Appendix 4 are derived approximate values (within a few centimeters) for the horizontal and vertical positions of flighted balls as functions of time in the presence of air resistance. Knowledge of the entire trajectory allows modeling of impacts with rising or falling balls at "head height", and also aids in understanding of the differences between balls played by adult and youth players. If the drag factor for air resistance on the ball is $\beta$ and the mass of the ball is m, then the horizontal position is

$$d(t) \cong v_h(0)\,t\left(1 - \frac{1}{2}\frac{\beta}{m}v_h(0)\,t\right). \tag{9}$$

So, when $\beta = 0$, $d(t)$ is the same as for the vacuum case given in expression (7). For $\beta > 0$, the distance traveled is less than the vacuum case. As drag increases, the horizontal position of the ball advances less rapidly than in a vacuum. Drag slows down the horizontal speed of soccer balls substantially, especially when time-of-flight or "hang time" is relatively long. From expression (9) $v_h(0)$ can be calculated from measured time-of-flight and distance, when constants $\beta$ and $m$ are known. Thereafter, $v_h(t)$ can be computed for any time, $t$. With this information one can find the horizontal speed of the ball at head height, which is of immediate concern in the biomechanics of heading.

To find the time at which the ball is at head height during descent, however, one must examine the vertical component of the ball's trajectory. The vertical component of the trajectory of a ball of mass, $m$, launched from ground level and opposed by air resistance is given to good approximation by the following expressions, derived in Appendix 4, where $h^*$ is the peak height and $t^*$ is the time to peak height.

$$h(t) \cong h^* - \frac{1}{2}g(t-t^*)^2\left[1 + \frac{1}{6}\frac{\beta}{m}g(t-t^*)^2\right] \text{ for rising flight } (t \le t^*), \tag{10a}$$

and

$$h(t) \cong h^* - \frac{1}{2}g(t-t^*)^2\left[1 - \frac{1}{6}\frac{\beta}{m}g(t-t^*)^2\right] \text{ for falling flight } (t > t^*). \tag{10b}$$

During rising flight the force of air resistance aids gravity, and the term involving drag factor, $\beta$, adds to the effect of gravity. During falling flight the force of air resistance opposes gravity, and the term involving drag factor, $\beta$, subtracts from the effect of gravity.

The values of $h^*$ and $t^*$ in terms of fundamental constants are

$$h^* = \frac{1}{2} g(t^*)^2 \qquad (11)$$

and

$$t^* \cong \frac{v_v(0)}{g}\left[1 - \frac{1}{3}\frac{\beta}{mg} v_v^2(0)\right] \cong \frac{1}{2} t^{**}\left(1 - \frac{1}{48}\frac{\beta}{m} g(t^{**})^2\right), \qquad (12)$$

where $t^{**}$ is the time-of-flight or hang time. Expressions (11) and (12) mean that both $h^*$ and $t^*$, and in turn $h(t)$, can be determined from time-of-flight data and the known constants $m$, $g$, and β.

The value of β depends on the density of air, ρ, the drag coefficient for a sphere, $C_D$, and the cross-sectional area of the ball, $A$[33,34]. Specifically,

$$\beta = \frac{1}{2} \rho A C_D . \qquad (13)$$

Using standard literature values, β = 0.033 kg/sec for a standard size 5 ball. This same value was also confirmed experimentally by dropping balls of varying cross-sectional areas from measured heights such that they hit the ground at the same instant as did a reference low drag-to-weight object — a golf ball — that was dropped from a constant 4.92 m height (Appendix 5).

### Statistical Profile of Horizontal Ball Speeds in Actual Games

Expressions (9) and (10) give estimates of $d(t)$ and $h(t)$ for any ball kicked with initial velocity components $v_h(0)$ and $v_v(0)$. To determine the statistical distribution of horizontal speeds of potentially headable balls played in actual games, we charted time and distance data for high balls in tournament games of youth and adult players. An observer with a stopwatch determined time-of-flight. A spotter with many years of playing and coaching experience determined distance by marking start and finish positions of flighted balls on a scale drawing of the playing field. These data were substituted into expressions (9) and (12) for $d(t)$ and $t^{**}$ , which were solved for $v_h(0)$ and $v_v(0)$, after which the complete trajectory of each ball could be determined. The horizontal components of ball speed at head level (1 to 1.7 meters, depending on player age) were then computed.

In order to be counted in the statistical profile, a ball must have reached a peak height clearly greater than the height of the players on the field. Also to be counted, balls had to be played from the ground or from below the waist (not headed or thrown) and allowed to hit the ground or trapped below the waist. Such balls had trajectories such that they could have been headed. These included goal kicks, corner kicks, crosses, high clearances from the back third of the playing field, and lofted through-balls played to space in front of onrushing strikers. Games involving higher skill level teams of various ages played in the Indianapolis, Indiana area in the spring and summer of 1999 were studied. Six sets of games were analyzed, including games played by under 10 ½ year-old boys and girls, under 14 ½ year-old boys and girls, under 18 ½ year-old mature teens, and professional ("A" and "W" league) men and women. These data allowed realistic estimation of the complete range of trajectories of headable balls in games for each age level of players.

### Estimation of the Damping Factor for Ball–Head Impact

Energy dissipation by the ball subtly influences the motion of head and ball during impact and can dampen accelerations of head and ball during heading. The damping factor is related to the decrement in height of successive bounces of a soccer ball rebounding from a hard floor. The lesser height of each bounce reflects energy absorption at impact. As shown in Appendix 6, the damping factor, $\mu$, can be estimated from the formula

$$\mu \cong -\frac{1}{2}\frac{\sqrt{CPm}}{\pi}\ln\left(\frac{h_1}{h_0}\right), \tag{14}$$

where $C$ is the ball circumference, $P$ is the ball inflation pressure, $m$ is the mass of the ball, $h_1$ is the height of the first bounce, and $h_0$ is the height from which the ball was dropped initially. In our experiments $h_0$ = 4.92 meters, a value producing a velocity at impact similar to that expected for average headers. Balls were tested over a range of inflation pressures ranging from 0.3 to 1.1 atm, over which $\mu$ was relatively constant. The mean damping constant, $\mu$, determined by the method of Appendix 6 for ten commercial soccer balls was 13 ± 2 Nt/(m/sec). Since the influence of damping constant, $\mu$, upon head acceleration is small, a mid-range value of $\mu$ = 13 Nt/(m/sec) was utilized in all computations. Using this value for damping constant and official ball weights, computations of head acceleration could be made for a wide variety of scenarios spanning a range of player masses, player running speeds, ball velocities, and ball inflation pressures.

### Other Variables

Player weights and heights for boys and girls of various ages were estimated from a pediatric growth chart[25]. Legal ball weight, circumference, and the upper limit of ball inflation pressure were based upon the Laws of the Game[20]. Player velocity toward or away from the ball was taken in the range of 0 to 1 m/sec on the basis of everyday playing experience.

### Computational Methods

A Microsoft Excel spreadsheet program was used to compute head and ball positions, velocities, and accelerations on the basis of expressions (4) through (6). Parameters of special interest included ball pressure, speed, circumference, and mass. The effective mass of the player ranged from approximately 5% of body weight for exceedingly poor technique to 50% of body weight for expert technique. The average value of head acceleration during any single impact—$\int_0^{\Delta t} \ddot{x}_1(t)dt \big/ \Delta t$ for contact time $\Delta t$—was found for a wide variety of heading scenarios covering reasonable practical values expected in youth, teenage, and adult games. This average value during impact was that recorded in Monte Carlo simulations.

### Monte Carlo Methods

To determine the range of head accelerations experienced by adult players using a size 5 (adult) ball and for youth players using a size 4 ball, we performed Monte Carlo simulations of normal headers. Ball velocities and effective masses of players were selected at random from realistic sampling distributions for normal game conditions. For adult players, the effective mass was selected from a normal (Gaussian) distribution with mean 27 kg and standard deviation 8 kg. In this distribution, excellent technique by an average 70-kg adult is represented by an effective mass 1.0 standard deviation above the mean, 35 kg. The horizontal ball velocity was selected at random from a Gaussian distribution with mean 5.7 m/sec and standard deviation 2.2 m/sec. These parameters closely match the observed distribution of horizontal ball velocities in adults (Fig. 2). Simulation was performed for three ball inflation pressures: hard (1.1 atm, the legal upper limit), medium (0.6 atm, the legal lower limit), and soft (0.3 atm, a proposed safer, lower limit). Similar simulations for youth players were done as described in Results.

### Reference Values for Clearly Safe and Clearly Dangerous Accelerations

Reference values for clearly safe head accelerations were computed for body motions of everyday life including repeated jumping, head nodding, and side-to-side bending at the waist (Table 2). Repetitive motions were assumed to be sinusoidal in form with displacement $x = A\sin(\omega t)$, where $A$ represents one half the peak-to-peak amplitude of head motion, and $\omega = 2\pi$ times the frequency of oscillation in cycles per second. From this expression the velocity and acceleration of the head are $dx/dt = \omega A\cos(\omega t)$ and $d^2x/dt^2 = -\omega^2 A\sin(\omega t)$, from which the maximal acceleration in either direction is $\omega^2 A$. Values of $\omega^2 A$ so calculated were assumed to be normal and non-worrisome accelerations that the human body has evolved to withstand without ill effects. They are similar to head accelerations during normal childhood play and are unlikely to be of concern to health professionals or parents.

**TABLE 2**
**Maximal Head Accelerations during Safe Reference Activities***

| Activity | Frequency (Hz) | 1/2 max excursion, A (meters) | Peak Acceleration (m/sec$^2$) | Peak Acceleration ($g$) | Duration (sec) | HIC ($g^{2.5}$-sec) |
|---|---|---|---|---|---|---|
| Head nodding | 3.33 | 0.03 | 13.13 | 1.34 | 0.15 | 0.10 |
| at three | 1.00 | 0.20 | 7.90 | 0.81 | 0.50 | 0.09 |
| frequencies | 2.30 | 0.08 | 16.71 | 1.70 | 0.22 | 0.27 |
| Jumping | 1.60 | 0.14 | 14.15 | 1.44 | 0.31 | 0.25 |
| Rocking | 1.42 | 0.17 | 13.53 | 1.38 | 0.35 | 0.26 |
| Mean values | 1.93 | 0.12 | 13.08 | 1.34 | 0.31 | 0.19 |

* For motion $x = A\sin(\omega t)$, $dx/dt = \omega A\cos(\omega t)$, and $d^2x/dt^2 = -\omega^2 A\sin(\omega t)$; maximum acceleration is $-\omega^2 A$.

Reference values for clearly dangerous head accelerations were calculated on the basis of the HIC established by the U.S. Department of Transportation for assessment of motor vehicle safety, including the performance of automobile seat belts and motor cycle helmets[22,23,24]. This scale has been used in sports medicine to evaluate performance of American football helmets and bicycle helmets[36]. The HIC is computed as the product of the duration of the impact and the mean value of head acceleration during impact, raised to the 2.5 power:

$$HIC = \Delta t \left[ \int_0^{\Delta t} \ddot{x}_1(t)dt \Big/ \Delta t \right]^{2.5} . \tag{15}$$

When the acceleration is measured in $g$'s (1 $g$ = 9.8 m/sec$^2$), HIC values of 1000 or greater are taken as sufficient to produce serious, even life-threatening injury with a single blow to the head. Values progressively less than 1000 are assumed to be progressively less likely to produce injury. The safe, normal head motions just described have HIC values averaging 0.2 (Table 2).

### CONCLUSIONS

Long-term soccer players exhibit subtle signs of brain injury that cannot be explained by the low incidence of concussions resulting from contact with other players, goal posts, or the ground. Much more likely culprits are heading accidents, in which balls strike players unaware, as well as unlucky instances of normal heading, in which especially fast balls are headed with especially poor technique. In the present biomechanical study, brain accelerations during normal heading by adult players average less than 0.1% of accepted traumatic levels for a single impact. However, greater and more worrisome accelerations during heading can occur under certain game-like conditions produced by poor technique, heavy, over-inflated balls, rising balls, and accidental head–ball impacts. Heading under these conditions could conceivably explain the reported evidence of brain injury in selected players. Youth players are at greater risk because of their lower body mass and less experienced technique. These potentially dangerous conditions can be avoided by prudent changes in practice and match conditions, especially for young players, without detracting from the beauty and excitement of the game.

### ACKNOWLEDGEMENT

The author thanks summer research student, Ms. Amy Allegretti, for much helpful technical assistance in this project.

## APPENDICES

### Appendix 1: Effective Mass of the Player

| Symbol | Definition | Units or value |
|---|---|---|
| $F_x$ | Horizontal force applied to head of player by ball | Newtons |
| L | Body length | meters |
| m | Mass of player | kg |
| m´ | Effective mass of player | kg |
| ρ(h) | Axial density of human body per unit length as a function of height, h | kg/m |
| θ | Angle of forward lean | Radians |
| $\ddot{\theta}$ | Angular acceleration of the player's body | Rad/sec$^2$ |
| $\ddot{x}$ | Acceleration of player's head in the x-dimension as a result of impact with the ball | m/sec$^2$ |

The effective mass, *m´*, of the player is that mass which would require the same horizontal force to accelerate in the x-dimension as does the total player in two or three dimensions. Here we assume temporarily excellent heading technique, in which the neck is strong and rigid and the head does not wobble. Consider first the grounded player sketched in text Fig. 1, whose plant foot is fixed, whose body is free to pivot, and who leans into the shot with angle θ. During contact θ will change by a relatively small value, in keeping with soccer playing experience. The horizontal force component, $F_x$, is applied by the ball to the player's head. By definition

$$m' = \frac{F_x}{\ddot{x}} = \frac{F_x}{L\ddot{\theta}\sin\theta} \ . \qquad \text{(A1.1)}$$

$F_x$ may be resolved into orthogonal components $F_x$ cos(θ) and $F_x$ sin(θ). For the grounded player $F_x$ cos(θ), which is parallel to the spine, does not induce body motion, because the spine is incompressible and the cleated boot prevents motion along this axis. Angular acceleration by $F_x$ sin(θ) is unconstrained, however. Our strategy is to find a second expression for $\ddot{\theta}$ from Newton's second law and substitute into A1.1 to determine m´. Integrating force = mass x acceleration for all small segments of mass along the length of the player's body and assuming that the angle of forward lean, θ, changes only a small amount during impact, we have

$$F_x \sin\theta = \ddot{\theta}\int_0^L h\,\rho(h)dh \ . \qquad \text{(A1.2)}$$

Suppose, for example that $\rho(h)$ is a constant value, $\rho$; i.e., we model the player for simplicity as a slab (a more reasonable assumption than it may first appear). Then,

$$F_x \sin\theta = \ddot{\theta}\,\rho\int_0^L h\,dh = \frac{1}{2}\ddot{\theta}\,\rho L^2 , \qquad \text{(A1.3)}$$

so that

$$\ddot{\theta} = \frac{2F_x \sin\theta}{\rho L^2}\,. \tag{A1.4}$$

Substituting A1.4 into A1.1 and noting $\rho L = m$, the effective mass for the slab model is

$$m' = \frac{m}{2\sin^2\theta}\,. \tag{A1.5}$$

Here, when θ = 0, the player is horizontal, and theoretically, $m' = \infty$, because of the ground anchor. When θ = 90° $m' = m/2$, which fits with the expected leverage if all body mass were located at the center of mass of the slab.

We can also compute the effective mass of a player executing a jumping header for the slab model of the body. Now the $F_x \cos(\theta)$ force component induces downward translation of the slab along the θ axis because there is no retarding contact with ground. The $F_x \sin(\theta)$ force component induces rotation about the center of mass of the slab. The effective mass retarding acceleration of the slab in the x-dimension is given by

$$m' = \frac{F_x}{\ddot{x}_{translational} + \ddot{x}_{rotational}}\,. \tag{A1.6}$$

By deduction from text Fig. 1 in the absence of a ground anchor, the x-component of translational acceleration is

$$\ddot{x}_{translational} = \frac{F_x \cos^2\theta}{m} \tag{A1.7}$$

The x-component of rotational acceleration is

$$\ddot{x}_{rotational} = \frac{L}{2}\ddot{\theta}\sin\theta, \tag{A1.8}$$

and $\ddot{\theta}$ can be found from

$$F_x \sin\theta = 2\ddot{\theta}\rho\int_0^{L/2} h\,dh = \ddot{\theta}\rho\frac{L^2}{4} \tag{A1.9}$$

Solving A1.9 for $\ddot{\theta}$ and combining A1.6 through A1.8 yields

$$m' = \frac{m}{2\sin^2\theta + \cos^2\theta}\,. \tag{A1.10}$$

Thus when θ = 0, the player is horizontal, and $m' = m$, as expected for a perfect diving header with the player suspended in air. When θ = 90° $m' = m/2$, the same as for the anchored slab model, as expected. For a reasonable playing value of $\theta = \tan^{-1}(4)$, representing the good technique of a player striking the ball with slight forward lean, the effective mass is 0.53 for the grounded player model and 0.515 for the jumping player model. Hence for routine computation of excellent technique we can estimate $m' = m/2$ to represent a player whose neck is strong and stiff (perfect technique) at impact. If the neck is allowed to wobble at impact (extremely poor technique), the effective mass approaches the mass of the head only. Thus a range of effective masses between these limits can represent a range of techniques. In this way we have a simple mathematical model of the performance of a wide range of complex mechanical linkages of the actual human body in action.

How different from a slab is a person in terms of moment of inertia and effective mass? Using anatomically realistic estimates of cross-sectional areas of bone and water density in an adult human body (data not shown) and the definite integral A1.2, the author found an anatomically realistic effective mass that differed less than 5% from that of the slab model. In terms of moments of inertia, people are rather like slabs. They have above average mass in the abdomen near the body's center of mass, and below average mass elsewhere, which yields an overall effective mass much like that of a slab.

### Appendix 2: Model of a Soccer Ball

| Symbol | Definition | Units or value |
|---|---|---|
| ε | Small value | various |
| F | Force applied to ball by head | Newtons |
| k | Spring constant of soccer ball | Nt/m |
| μ, μ’ | Damping coefficient for energy absorption by ball | Nt/m/sec |
| P | Pressure inside ball | atmospheres |
| π | Circle ratio | 3.14159 |
| R | Radius of ball | meters |
| r | Radius of indented area on ball | meters |
| θ | Angle between axis of ball and rim of indentation | radians |
| u | Indentation of ball | meters |

Text Fig. 9 shows a flat indentation caused by impact of the head or instep of the foot contacting a spherical soccer ball of original radius $R$ inflated to pressure $P$ greater than ambient atmospheric pressure. The radius of the indentation is r, defining angle θ. The direction of the indenting force defines the x-axis of head–ball interaction. We seek the relationship between the amount of indentation, u, the rate of indentation, *du/dt*, and the force, $F$, generated between head and ball. First consider the outward force caused by pressure inside the ball

$$F_1 = (P+\varepsilon)\pi r^2 \approx P\pi r^2, \tag{A2.1}$$

where ε is the small increase in pressure caused by flattening of the ball. Here

$$r^2 + (R-u)^2 = R^2, \tag{A2.2}$$

which after rearranging becomes

$$r^2 = 2Ru\left(1-\frac{u}{2R}\right) \approx 2Ru \tag{A2.3}$$

for small indentations, which is reasonable for soccer headers. Combining A2.3 and A2.1 with offsetting errors gives

$$F_1 \cong 2\pi PRu = ku, \tag{A2.4}$$

where $k = 2\pi PR$ is a classical Hook’s Law spring constant for the ball. Thus the ball pushes back on the impacting head or foot like a simple Hook’s Law spring.

Compression of air inside the ball does not absorb energy but returns it completely during recoil. Energy is lost, however, during bending or buckling of the ball’s cover at the edge of the indentation zone. This process may be modeled by introducing a small cover-buckling force proportional to the circumference of the buckling zone and the angular rate of buckling,

$$F_2 = \mu' \cdot 2\pi r \cdot \frac{d\theta}{dt}. \tag{A2.5}$$

Here it is helpful to introduce a change of variable from θ to *u*.

$$r = R\sin\theta \quad and \quad dr = R\cos\theta d\theta ,$$

or

$$d\theta = \frac{1}{R\cos\theta}dr = \frac{1+\varepsilon}{R}dr \qquad \text{(A2.6)}$$

for small indentations for which cosθ is close to 1. Similarly from A2.3, taking

$$r^2 = 2Ru(1-\varepsilon'),$$

$$du = \frac{1}{R(1-\varepsilon')}rdr ,$$

or

$$dr = \frac{R(1-\varepsilon')}{r}du \qquad \text{(A2.7)}$$

Combining A2.5 through A2.7

$$F_2 \cong 2\pi\mu' \frac{du}{dt} \equiv \mu \frac{du}{dt} \qquad \text{(A2.8)}$$

to a good approximation with offsetting errors. The constant μ is a classical damping coefficient. Thus we can model mathematically the total force acting between head and ball due to the action of both the pressure inside the ball and the buckling of its cover as

$$F = F_1 + F_2 = ku + \mu\frac{du}{dt} \ . \qquad \text{(A2.9)}$$

### Appendix 3: Equations of Motion for Impact

| Symbol | Definition | Units or value |
|---|---|---|
| A, B, C, D, E, a, b, c | Defined constants | various |
| e | Base of natural logarithms | 2.718 |
| F | Force | Newtons |
| k | Spring constant of ball | Nt/m |
| m | Mass of ball or effective mass of player | kg |
| $\mu$ | Damping coefficient for energy absorption by ball | Nt/(m/sec) |
| t | Time | sec |
| u | Indentation of ball by head | meters |
| $\omega$ | Angular frequency | radians/sec |
| x | Distance | meters |
| $\dot{x}$ | Velocity | m/sec |
| $\ddot{x}$ | Acceleration | m/sec$^2$ |
| Subscripts: | | |
| 1 | Player | |
| 2 | Ball | |

Motion during head–ball impact in the horizontal dimension is reckoned in the coordinate system of text Fig. 10. $x_1$ is the position of the leading edge of the head. $x_2$ is the position of the leading edge of the un-indented ball, one radius from its center. To minimize complexity of notation, the origin of the coordinate system is chosen such that $x_1 = x_2 = 0$ at the onset of impact when $t = 0$.

#### *Identities*

Ball indentation $u = x_1 - x_2$ during impact; $u = 0$ otherwise (A3.1)

By definition and the result of Appendix 2, the force of the ball acting on the head is

$$F_1 = -ku - \mu \frac{du}{dt} . \qquad \text{(A3.2)}$$

The force of the head acting on the ball is

$$F_2 = +ku + \mu \frac{du}{dt} . \qquad \text{(A3.3)}$$

From Newton's second law ($F = ma$), A3.2, and A3.3

$$m_1 \ddot{x}_1(t) = -m_2 \ddot{x}_2(t) \ . \qquad \text{(A3.4)}$$

Our strategy is to solve for $x_2$, $u$, and $\frac{du}{dt}$ in terms of $x_1$ and the known initial velocities of head and ball, $\dot{x}_1(0)$ *and* $\dot{x}_2(0)$, and to substitute into the equation of motion for the head, which is the mass $m_1$. To find $x_2$ in terms of $x_1$, note

$$\dot{x}_1(t) - \dot{x}_1(0) = \int_0^t \ddot{x}_1(t)dt \tag{A3.5}$$

$$\dot{x}_2(t) - \dot{x}_2(0) = \int_0^t \ddot{x}_2(t)dt = -\frac{m_1}{m_2}\int_0^t \ddot{x}_1(t)dt\,. \tag{A3.6}$$

Eliminating the integral from A3.5 and A3.6 and rearranging,

$$\dot{x}_2(t) = \dot{x}_2(0) - \frac{m_1}{m_2}\left(\dot{x}_1(t) - \dot{x}_1(0)\right). \tag{A3.7}$$

Substituting A3.7 into $\frac{du}{dt} = \frac{dx_1}{dt} - \frac{dx_2}{dt}$ and rearranging,

$$\frac{du}{dt} = \left(1 + \frac{m_1}{m_2}\right)\frac{dx_1}{dt} + \left(\dot{x}_2(0) + \frac{m_1}{m_2}\dot{x}_1(0)\right). \tag{A3.8}$$

In turn,

$$\int_0^u du = \left(1 + \frac{m_1}{m_2}\right)\int_0^{x_1} dx_1 + \left(\dot{x}_2(0) + \frac{m_1}{m_2}\dot{x}_1(0)\right)\int_0^t dt \tag{A3.9}$$

or

$$u = \left(1 + \frac{m_1}{m_2}\right)x_1 + \left(\dot{x}_2(0) + \frac{m_1}{m_2}\dot{x}_1(0)\right)t\,. \tag{A3.10}$$

#### *Equations of Motion*

The equation of motion for the head during impact is $F_1 = m_1\ddot{x}_1$, or

$$m_1\ddot{x}_1 - F_1 = 0 = m_1\ddot{x}_1 + ku + \mu\frac{du}{dt}\ . \tag{A3.11}$$

Substituting A3.8 and A3.10 into A3.11 and rearranging yields a governing differential equation for the position, $x_1$, of the head (or by an entirely similar route for the position of the ball, $x_2$) during impact. The second-order differential equations for the motions of both head and ball have the form

$$A\ddot{x} + B\dot{x} + Cx + Dt + E = 0, \tag{A3.12}$$

where

$$A = m_1 m_2$$
$$B = \mu(m_1 + m_2)$$
$$C = k(m_1 + m_2)$$
$$D = -k\big(m_1\dot{x}_1(0) + m_2\dot{x}_2(0)\big)$$
$$E = -\mu\big(m_1\dot{x}_1(0) + m_2\dot{x}_2(0)\big).$$

The solution to A3.12 is

$$x = a\sin(\omega t)e^{-bt} + ct \tag{A3.13}$$

with

$$\dot{x} = a\omega\cos(\varpi t)e^{-bt} - ab\sin(\omega t)e^{-bt} + c \tag{A3.14}$$

and

$$\ddot{x} = -a\big[(\omega^2 - b^2)\sin(\omega t) + 2\omega b\cos(\omega t)\big]e^{-bt} \tag{A3.15}$$

where

$$a = \frac{\dot{x}(0) - c}{\omega}$$

from A3.14 at $t = 0$, and by using the method of undetermined coefficients (substituting A3.13 – A 3.15 into A3.12 and equating sine and cosine terms),

$$b = \frac{B}{2A},$$

$$c = \frac{-E}{B}, \quad \text{and}$$

$$\omega^2 = \frac{C}{A} - \frac{1}{4}\frac{B^2}{A^2} .$$

The differential equations for player and ball motion differ only in the initial conditions. For $x_1$ at $t = 0$, $\dot{x} = \dot{x}_1(0)$, the initial velocity of the head toward the ball. For $x_2$ at $t = 0$, $\dot{x} = \dot{x}_2(0)$, the initial velocity of the ball toward the head.

Thus it is possible to compute A3.14 through A3.16 to obtain the position, velocity, and acceleration of either head or ball using constants

$$a_1 = \frac{\left(\dot{x}_1(0) - \dot{x}_2(0)\right)m_2}{\omega\left(m_1 + m_2\right)} \tag{A3.16}$$

$$a_2 = \frac{-\left(\dot{x}_1(0) - \dot{x}_2(0)\right)m_1}{\omega\left(m_1 + m_2\right)} = -\frac{m_1}{m_2}a_1 \tag{A3.17}$$

$$b = \mu\frac{m_1 + m_2}{2m_1 m_2} \tag{A3.18}$$

$$c = \frac{m_1\dot{x}_1(0) + m_2\dot{x}_2(0)}{m_1 + m_2} \tag{A3.19}$$

$$\omega = \sqrt{k\frac{m_1 + m_2}{m_1 m_2} - \left(\frac{1}{2}\frac{\mu(m_1 + m_2)}{m_1\, m_2}\right)^2}\ . \tag{A3.20}$$

### Appendix 4: Soccer Ball Trajectories with $v^2$ Air Resistance

| Symbol | Definition | Units or value |
|---|---|---|
| A | Cross-sectional area of ball | $m^2$ |
| β | Air resistance coefficient | $Nt/(m^2/sec^2)$ |
| c | Constant of integration | m/sec |
| $C_D$ | Drag coefficient | dimensionless |
| d | Distance (horizontal component of ball trajectory) | meters |
| e | Base of natural logarithms | 2.718 |
| F | Force | Newtons |
| g | Gravitational acceleration of falling body at Earth's surface | $9.80\ m/sec^2$ |
| h | Vertical component of ball trajectory | meters |
| ln | Natural logarithm | dimensionless |
| m | Mass of soccer ball | kg |
| ρ | Density of air | $kg/m^3$ |
| t | Time | sec |
| t* | Time to peak height of ball | sec |
| t** | Hang time for ball to return to earth | sec |
| τ | Time difference | sec |
| v | Velocity | m/sec |
| Subscripts: | | |
| h | Horizontal component | |
| v | Vertical component | |

#### *Horizontal Component of Ball Flight d(t)*

Classically air resistance or drag is proportional to velocity squared:

$$F_{drag} = -\frac{1}{2}\rho A C_D \cdot v^2 \equiv -\beta \cdot v^2$$

for a given sized ball. In the horizontal dimension there is no effect of gravity, and the equation of motion for the ball is

$$m\frac{dv}{dt} = -\beta v_h^2 . \quad \text{(A4.1)}$$

Rearranging and integrating

$$\int_{v_h(0)}^{v_h} \frac{dv_h}{v_h^2} = -\frac{\beta}{m}\int_0^t dt , \quad \text{(A4.2)}$$

which leads to

$$v_h = \frac{v_h(0)}{1 + v_h(0)\frac{\beta}{m}t}, \tag{A4.3}$$

where $v_h(0)$ refers to the initial horizontal component of ball velocity at time 0.

$$\text{Distance } d(t) = \int_0^t v_h dt = \frac{m}{\beta}\ln\left(1 + v_h(0)\frac{\beta}{m}t\right). \tag{A4.4}$$

To better appreciate this result intuitively, consider the case when β is small, which is true for balls in air. Using the series expansion for the natural logarithm, $\ln(1+x) \approx x - \frac{x^2}{2} + \ldots$, one obtains expression (9) in the text, which is

$$d(t) \cong v_h(0)t\left(1 - \frac{1}{2}\frac{\beta}{m}v_h(0)t\right). \tag{A4.5}$$

The distance traveled is less than the vacuum case by an amount depending on the drag-to-mass ratio, initial velocity, and time of flight.

#### *Vertical Component of Ball Flight h(t)*

Let drag coefficient $\beta = +\frac{1}{2}\rho A C_D$ for rising flight and $\beta = -\frac{1}{2}\rho A C_D$ for falling flight.

Taking upward velocity as positive, we can write vertical ball acceleration = force/mass as

$$\frac{dv_v}{dt} = \frac{-mg - \beta v_v^2}{m} = -g\left(1 + \frac{\beta}{mg}v_v^2\right). \tag{A4.6}$$

By the discontinuous definition of β, the $v^2$ drag term adds to the effect of gravity in slowing the ball's ascent, but opposes the effect of gravity in slowing the ball's descent. Separating the variables and integrating,

$$\int \frac{dv_v}{1 + \frac{\beta}{mg}v_v^2} = -\int g dt. \tag{A4.7}$$

Since the effect of air resistance is small for soccer balls, we can simplify the integration by introducing the approximation $\frac{1}{1+x} \approx 1 - x$ for small $x$. Then we have

$$\int\left(1-\frac{\beta}{mg}v_v^2\right)dv \cong -\int g\,dt \qquad \text{(A4.8)}$$

or

$$v_v - \frac{1}{3}\frac{\beta}{mg}v_v^3 = -gt + c \qquad \text{(A4.9)}$$

From the boundary condition that $v_v = v_v(0)$ at $t = 0$, one can evaluate the constant of integration,

$$c = v_v(0)\left(1-\frac{1}{3}\frac{\beta}{mg}\left(v_v(0)\right)^2\right),$$

and from the condition that $v = 0$ at $t = t^*$, the time to peak height, $c = gt^*$. In turn,

$$t^* = \frac{v_v(0)}{g}\left(1-\frac{1}{3}\frac{\beta}{mg}\left(v_v(0)\right)^2\right).$$

Introducing the change of variable $\tau = t - t^*$, we have the more compact expression

$$v_v - \frac{1}{3}\frac{\beta}{mg}v_v^3 = -g(t-t^*) = -g\tau. \qquad \text{(A4.10)}$$

Now for small values of $\beta$, $v_v \approx -g\tau$, so we can write

$$v_v \cong -g\tau + \frac{1}{3}\frac{\beta}{mg}(-g\tau)^3 = -g\tau - \frac{1}{3}\frac{\beta}{m}g^2\tau^3. \qquad \text{(A4.11)}$$

To obtain approximate height of a soccer ball in flight one may simply integrate as follows.

$$h(\tau) = \int_{-t^*}^{\tau} v_v(\tau)d\tau \cong \left(-\frac{1}{2}g\tau^2 - \frac{1}{12}\frac{\beta}{m}g^2\tau^4\right]_{-t^*}^{\tau} \qquad \text{(A4.12)}$$

The maximum height $h^*$ occurs when $t = t^*$ or $\tau = 0$. In particular, from A4.12

$$h^* \cong \frac{1}{2}g(t^*)^2 - \frac{1}{12}\frac{\beta}{m}g^2(t^*)^4.$$

Considering that

$$\int_{-t^*}^{\tau} v_v(\tau)d\tau = \int_{-t^*}^{0} v_v(\tau)d\tau + \int_{0}^{\tau} v_v(\tau)d\tau,$$

we have

$$h(\tau) = \int_{-t*}^{\tau} v_v(\tau) d\tau = \int_{-t*}^{0} v_v(\tau) d\tau + \int_{0}^{\tau} v_v(\tau) d\tau \cong h* - \frac{1}{2} g\tau^2 \left( 1 + \frac{1}{6} \frac{\beta}{m} g\tau^2 \right), \qquad \text{(A4.13)}$$

where, as before, the sign of constant, β, is positive for rising flight (τ < 0) and negative for falling flight (τ > 0).

### Appendix 5: Dropped Ball Experiments to Determine Drag Factor, β

Suppose that reference ball, 1, having very little air resistance (e.g., a golf ball), and test ball, 2, having significant air resistance (e.g., a soccer ball), are dropped at the same instant in time from heights $h_1 > h_2$ above the ground plane. On successive trials let $\Delta h = h_1 - h_2$ be adjusted such that the two balls strike the ground at the same instant, as judged by sight and sound of an observer at ground level who acts as a "null detector". The force of air resistance is proportional to the square of the velocity. Hence the equation of motion for each ball is

$$m\frac{dv}{dt} = mg - \beta v^2 \quad or \quad \frac{dv}{dt} = g\left(1 - \frac{\beta}{mg}v^2\right). \tag{A5.1}$$

Separating the variables,

$$\int_0^v \frac{dv}{1 - \frac{\beta}{m}v^2} = \int_0^t g\,dt, \tag{A5.2}$$

and taking $\frac{1}{1-x} \approx 1 + x$ for the small $x$ term involving β, as in A4.6 – A4.8, and integrating, we have

$$v + \frac{1}{3}\frac{\beta}{mg}v^3 = gt. \tag{A5.3}$$

Proceeding as in Appendix 4 (A4.10 – A4.12) with $v \cong gt$ for cases of small β, the downward velocity

$$v \cong gt - \frac{1}{3}\frac{\beta}{m}g^2t^3,$$

and the total vertical distance traveled in time-of-flight, $t$, is

$$h = \int_0^t v\,dt \cong \frac{1}{2}gt^2 - \frac{1}{12}\frac{\beta}{m}g^2t^4, \tag{A5.4}$$

which is a quadratic in $t^2$.

To explore the "null" test condition in which $t_1 = t_2$ for balls 1 and 2, we can solve the forgoing expression for $t$, equate the times for balls 1 and 2, and then solve for β. To simplify notation let $u = \frac{1}{2}gt^2$; then

$h \cong u - \frac{1}{3}\frac{\beta}{m}u^2$, for which the quadratic formula gives $u = \dfrac{1 \pm \sqrt{1 - \frac{4}{3}\frac{\beta}{m}h}}{\frac{2}{3}\frac{\beta}{m}}$ .

Using the series expansion $\sqrt{1+\varepsilon} \cong 1 + \frac{1}{2}\varepsilon - \frac{1}{8}\varepsilon^2$ for $\varepsilon << 1$ for the square root term, which is true for soccer balls, since β is small, and simplifying we have

$$u \cong h + \frac{1}{3}\frac{\beta}{m}h^2. \qquad \text{(A5.5)}$$

If test ball 2 is dropped from lesser height, $h_2$, so that it hits the ground at exactly the same time as reference ball 1 with much smaller or negligible β, then $t_1 = t_2$ , so

$$u_1 = u_2 = h_1 + \frac{1}{3}\frac{\beta_1}{m_1}h_1^2 = h_2 + \frac{1}{3}\frac{\beta_2}{m_2}h_2^2. \qquad \text{(A5.6)}$$

For an ideal reference ball $\beta_1 = 0$, and

$$h_1 - h_2 = \Delta h = \frac{1}{3}\frac{\beta_2}{m_2}h_2^2 \;,$$

from which

$$\beta_2 = \frac{3\Delta h m_2}{h_2^2}$$

in terms of the measured heights and the ball mass. For the case of $\beta_1 << \beta_2$ but not equal to zero a similar approach gives

$$\beta_2 = \frac{3\Delta h m_2}{h_2^2 - \Phi h_1^2} \;, \qquad \text{(A5.7)}$$

where we take advantage of the fact that air resistance is proportional to the cross sectional profile area, $A$, for similarly shaped objects, such that

$$\beta_1 = \frac{A_1}{A_2}\beta_2 \text{ , and } \Phi = \frac{A_1}{m_1}\Big/\frac{A_2}{m_2}.$$

### Appendix 6: Dropped Ball Experiments to Determine Damping Factor, μ

| Symbol | Definition | Units or value |
|---|---|---|
| a, b, c | Defined constants from Appendix 3 | various |
| e | Base of natural logarithms | 2.718 |
| F | Force | Newtons |
| g | Gravitational acceleration at the Earth's surface | 9.8 m/sec$^2$ |
| h | Height of ball above hard, flat surface | meters |
| k | Spring constant of ball | Nt/m |
| $m_2$ | Mass of ball | kg |
| μ | Damping coefficient for energy absorption by ball | Nt/(m/sec) |
| π | Circle ratio | 3.14159 |
| t | Time | sec |
| u | Indentation of ball by head | meters |
| ω | Angular frequency | radians/sec |
| x | Distance separating ball and ground (<0 during impact) | meters |
| $\dot{x}$ | Velocity of ball during impact | m/sec |
| Subscript: | | |
| o | Initial value at beginning of drop | |

Suppose a ball is dropped from initial height, $h_o$, onto a hard, flat surface and bounces to height $h^*$. The energy absorption by the ball, determined by the damping coefficient, μ, is related to the ratio of potential energy at the peak height of the first bounce to the original potential energy, which is equal to the height ratio, $h^*/h_o$. The mathematics of Appendix 3 can be used to determine μ from measurements of $h^*/h_o$, taking the "head" to be the Earth of infinite mass, and the ball to have an impact velocity, $v_2(0)$, determined by conservation of energy:

$$m_2 g h_0 = \frac{1}{2} m_2 (v_2(0))^2 .$$

From A3.14, during impact of the ball with the ground,

$$x_2 = a_2 \sin(\omega t) e^{-bt} + ct , \qquad \text{(A6.1)}$$

where, as is evident from A3.18 – A3.22, for infinite "head" mass, $m_1$ ,

$$c = 0, \quad b = \frac{\mu}{m_2}, \quad and \quad \omega \cong \sqrt{\frac{k}{m_2}} .$$

Let $t = 0$ at the beginning of impact, and let $t = t'$ at the end of impact. At these times the ball-ground separation, $x_2 = 0$. Hence at $t'$, after all energy absorption has taken place, from A6.1 we have $t' = \pi/\omega$ exactly. By differentiation of A6.1

$$\dot{x}_2(t') = [a_2 \omega \cos(\omega t') + a_2 b \sin(\omega t')] e^{-bt'} = -a_2 \omega e^{-\pi b/\omega} , \qquad \text{(A6.2)}$$

and if μ = $b$ = 0, $\dot{x}_2(t') = -a_2\omega = v_2(0)$, as expected. Now to solve for μ (or $b$) in terms of $h^*/h_o$ we equate potential and kinetic energies.

$$m_2 g h^* = \frac{1}{2} m_2 \left(\dot{x}_2(t')\right)^2 \qquad \text{(A6.3)}$$

Combining A6.2 and A6.3,

$$h^* = \frac{1}{2g} a_2^2 \omega^2 e^{-2b\pi/\omega} \qquad \text{(A6.4)}$$

If $b$ = 0 there is no damping and perfect energy conservation, so

$$h^* = h_o = \frac{a_2^2 \omega^2}{2g} \ , \qquad \text{(A6.5)}$$

and so

$$h^* = h_o e^{-2b\pi/\omega} \ . \qquad \text{(A6.6)}$$

Taking logs and substituting $b = \mu/m_2$ and $\omega \cong \sqrt{\frac{k}{m_2}}$ , we have

$$-\ln\left(\frac{h^*}{h_o}\right) = \frac{2b\pi}{\omega} \cong \frac{2\mu\pi}{m_2}\sqrt{\frac{m_2}{k}} \ , \qquad \text{(A6.7)}$$

and

$$\mu \cong -\frac{1}{2}\frac{\sqrt{km_2}}{\pi}\ln\left(\frac{h^*}{h_o}\right) \ . \qquad \text{(A6.8)}$$

Since constant $k$ = ball circumference × inflation pressure is easily measured for a soccer ball, expression A6.8 provides an experimental route to measuring the damping factor, $\mu$, from the ratio of bounce height to launch height. The approximation in A6.8 is good to about 1 part in 1000.
